# Science Educational Outreach Programs That Benefit Students and Scientists

**DOI:** 10.1371/journal.pbio.1002368

**Published:** 2016-02-04

**Authors:** Greg Clark, Josh Russell, Peter Enyeart, Brant Gracia, Aimee Wessel, Inga Jarmoskaite, Damon Polioudakis, Yoel Stuart, Tony Gonzalez, Al MacKrell, Stacia Rodenbusch, Gwendolyn M. Stovall, Josh T. Beckham, Michael Montgomery, Tania Tasneem, Jack Jones, Sarah Simmons, Stanley Roux

**Affiliations:** 1 Department of Molecular Biosciences, The University of Texas at Austin, Austin, Texas, United States of America; 2 College of Natural Sciences, The University of Texas at Austin, Austin, Texas, United States of America; 3 Freshman Research Initiative, The University of Texas at Austin, Austin, Texas, United States of America; 4 Texas Institute for Discovery Education in Science, The University of Texas at Austin, Austin, Texas, United States of America; 5 Institut Pasteur, Unité de Génétique des Biofilms, Département de Microbiologie, Paris, France; 6 Austin Independent School District, Austin, Texas, United States of America; 7 Howard Hughes Medical Institute, Chevy Chase, Maryland, United States of America

## Abstract

Both scientists and the public would benefit from improved communication of basic scientific research and from integrating scientists into education outreach, but opportunities to support these efforts are limited. We have developed two low-cost programs—"Present Your PhD Thesis to a 12-Year-Old" and "Shadow a Scientist”—that combine training in science communication with outreach to area middle schools. We assessed the outcomes of these programs and found a 2-fold benefit: scientists improve their communication skills by explaining basic science research to a general audience, and students' enthusiasm for science and their scientific knowledge are increased. Here we present details about both programs, along with our assessment of them, and discuss the feasibility of exporting these programs to other universities.

## Abstract 2

The education outreach programs “Present Your PhD Thesis to a 12-Year-Old” and “Shadow a Scientist” provide opportunities for scientists to improve science communication skills and for students to learn about research.

## Introduction

Both the scientific community and the general public stand to benefit from improved communication of basic scientific research [1–3]. Having a science-literate, or even sympathetic, public has major implications for both the health of our society and for the climate for public funding of research. There is also a need to draw scientists into education outreach and provide for their professional development in science communication and education [4–6]. Experience teaching science has been shown to improve skills in generating testable hypotheses and in designing experiments [7], and it correlates with higher rates of publication [8]. The National Science Foundation (NSF) explicitly supports science communication efforts through its Broader Impacts, a mandatory section of all grant proposals, which requires that each project have, in some way, the “potential to benefit society and contribute to the achievement of specific, desired societal outcomes,” which includes encouraging science education outreach (NSF Grant Proposal Guide, Office of Management and Budget [OMB] Control Number 3145–0058). Despite this NSF mandate, there still are very few cost-effective models that provide training in science communication to graduate students and research scientists while benefiting the community [9–11].

Engaging graduate students and research scientists in outreach activities that provide science education for students between the ages of 5 and 18 years old (K–12) is a direct way of increasing young students’ awareness of ongoing scientific research. It is also a valuable opportunity for scientists to improve their science communication skills. Inquiry-based (as opposed to content-based) teaching in particular has been shown to result in both higher content retention and more positive attitudes toward the subject matter on the part of the students [12,13]. Graduate students and research scientists at the University of Texas at Austin initiated two novel educational outreach programs that combine training in science communication with outreach to area middle schools: “Present Your PhD Thesis to a 12-Year-Old” and “Shadow a Scientist.” Both programs provide a venue and structure for scientists to convey their current research to middle school students through direct interaction and mentorship. Both also emphasize an inquiry-based approach to science by providing actual examples of generating and testing hypotheses (in the case of “Present Your PhD Thesis to a 12-Year-Old”) and open-ended hands-on explorations (“Shadow a Scientist”). By participating in these programs, scientists become more involved in community outreach and gain experience in communicating the basic concepts and importance of their research to a general audience.

Both programs communicate current research to middle school students. Our assessment of these programs revealed a 2-fold benefit: the scientists improve their communication skills by explaining basic science research to a general audience, and the students’ enthusiasm for science and their scientific knowledge are increased. Here we describe these two relatively small, scientist-initiated and scientist-operated programs, present the results from our assessment of the programs, and discuss the feasibility of exporting them to other universities.

## Shadow a Scientist

The Shadow a Scientist program matches two middle school students with a scientist for a 2-hour tour of the scientist’s lab and experiments in progress. The Freshman Research Initiative, a program at the University of Texas at Austin that engages undergraduates in real research during their freshman year (http://cns.utexas.edu/fri), sponsors this outreach program. Shadowing a scientist gives middle school students the opportunity to visit laboratories on a university campus and interact with scientists. The middle school students—who have come from private, public, and home schools—visit one scientist who is selected based on their personal interests. On a typical visit, the students meet with the scientist and are introduced to his or her research. The students ask questions about research, do hands-on tasks in the lab, and are shown lab equipment.

The goals of the program include providing the opportunity for young students to cultivate their interest in science, allowing them to experience firsthand what a scientist does on a daily basis and providing graduate students and scientists the opportunity to explain their research and practice and improve their science communication skills. The logistics for this program are the responsibility of the program director and involve recruiting students by contacting administrators or teachers at local middle schools as well as coordinating with both parents and scientists to find a specific date that a student will shadow a particular scientist. Middle school students complete an application, which is used to schedule the student’s visit to the university on a first-come, first-served basis and to match students with scientists in an area of research that interests them. Because 10–18 students participate on any given day, undergraduate students engaged in research participate as volunteers to be student escorts, providing an additional outlet for undergraduate students to participate in educational outreach. To ensure participant safety, students are required to wear appropriate lab clothing, i.e., long pants or a long skirt and closed-toed shoes. Each student must also complete a liability form before the shadowing day. Additional information, as well as a sample PDF application and liability form, can be found at http://cns.utexas.edu/fri/outreach/164-freshman-research-initiative/outreach/633-shadow-a-scientist-program.

In addition to providing scientists with the opportunity to hone their science education outreach skills, short-duration summer science, technology, engineering and math (STEM) projects have other documented positive outcomes for participating students and scientists [14,15]. Since 2011, Shadow a Scientist has hosted a total of 503 middle school students at no charge. Forty-seven scientists, including graduate students, postdoctoral fellows, and professors from diverse disciplines, have hosted students. In addition to these traditional types of scientists, many of the scientists participating in the program have been “research educators,” a position for PhD research scientists teaching Freshman Research Initiative undergraduate research courses. This program provides the opportunity for research scientists to improve their science communication skills while sharing their research interests with young students and engaging in community outreach.

## Present Your PhD Thesis to a 12-Year-Old

In “Present Your PhD Thesis to a 12-Year-Old,” graduate student researchers in STEM fields present a simplified version of their PhD thesis in middle school classrooms or community centers. This program gives emerging scientists the opportunity to communicate their discoveries to middle school students and fuel students’ curiosity and enthusiasm for science. Importantly, the program provides a framework for graduate students to participate in community outreach and develop their science communication skills at an early stage in their science careers.

Thus, a key goal of this program is to provide an avenue for graduate students to improve their science communication skills. Graduate students who join this program start out as “presenters” and initially learn how to present their PhD thesis to a broad audience. Presenters develop a visual, interactive presentation on their PhD thesis that can be easily understood by middle school students. Each presentation is approximately 20 minutes, and an engaging, interactive format is encouraged. Previous exemplary presentation samples are available for viewing, and new presentations are carefully vetted during practice sessions aimed at helping the new presenter develop a high-quality presentation. During practice talks for each new presenter, there is an audience of three or more experienced graduate student presenters from the program. This setting provides ample opportunities for the new presenters to meet the outreach group and to benefit from feedback from more experienced presenters with multiple perspectives based on lessons they have learned and feedback from their previous presentations.

Another goal of this outreach program is to provide a structure that enables graduate students to gain experience in initiating and developing ongoing relationships in their community. Therefore, the program relies on some “presenters” becoming “organizers” who initiate and facilitate presentation series within their communities. Organizers are autonomous and are each free to develop a lecture series with local educators in a manner that fits their schedules. Thus, one organizer might visit their community venue(s) several times a month, while another might visit a couple of times a semester. Each organizer initially introduces his or her lecture series to the students near the beginning of the school year by giving a broad presentation that explains what graduate school and a PhD thesis is and describes his or her own experience as a graduate student and scientist.

Organizers often initiate their first presentation series based on a personal connection with a teacher or administrator. Organizers often choose to reach out to a particular school where most of the students are from backgrounds underrepresented in the sciences. They also receive requests for presentations from educators who find out about the program from our website. The simple structure and distributed responsibility of this program allows for scalability and continuity of the program even without a full-time administrator and despite turnover as graduate students leave the university.

Typically presentations lead to many questions from the students. After the presentations, students complete forms with a rubric to provide written feedback to the presenter. A rubric is potentially an important tool for improving the ability of graduate students to explain their research [16]. Our rubric allows graduate students to receive both quantitative and free-form assessment of various aspects of their presentation. Feedback forms are designed to be engaging, so middle school students are not quizzed on the material presented but instead are asked to rate the presenter on enthusiasm and accessibility and suggest improvements (S1 Fig).

Over the past four years, 36 graduate students, mainly from biology disciplines (see examples of presentation titles), have presented at 11 educational venues in Austin and the surrounding rural communities (S2A and S2B Fig). Although the program was developed for middle school students, younger and older students have also participated. In the first four years, presentations were given to a total of 1,002 K–12 students (ages 5–18). The student audiences ranged from a small group of 12 at the Boys and Girls Club, to multiple classrooms of students at a private elementary school in auditorium presentations, to several classrooms at a public science magnet middle school in a series of presentations.

In addition to helping graduate students develop science communication skills, the program also offers potential benefits for students and teachers [17]. Students can benefit by learning about ongoing research, which adds to and complements science topics covered in the classroom. Students also become familiar with the ways research is designed and performed and learn firsthand about graduate school and science as a career path. Teachers can also benefit by bringing outside expertise into their science classroom that often leads to increased interest in related science topics covered in the teacher’s subsequent lessons. Additional information about the program and the participants as well as videos of past presentations can be found at http://www.utexas.edu/ogs/research/outreach/.

## Impact of Outreach Programs

Informal feedback from participants in both of these programs has been very positive. However, in order to quantitatively assess the effectiveness of these programs, during this past year we asked participating scientists and middle school students (ages 11–14) to voluntarily complete anonymous surveys aimed at assessing their outcomes.

**Fig 1:**
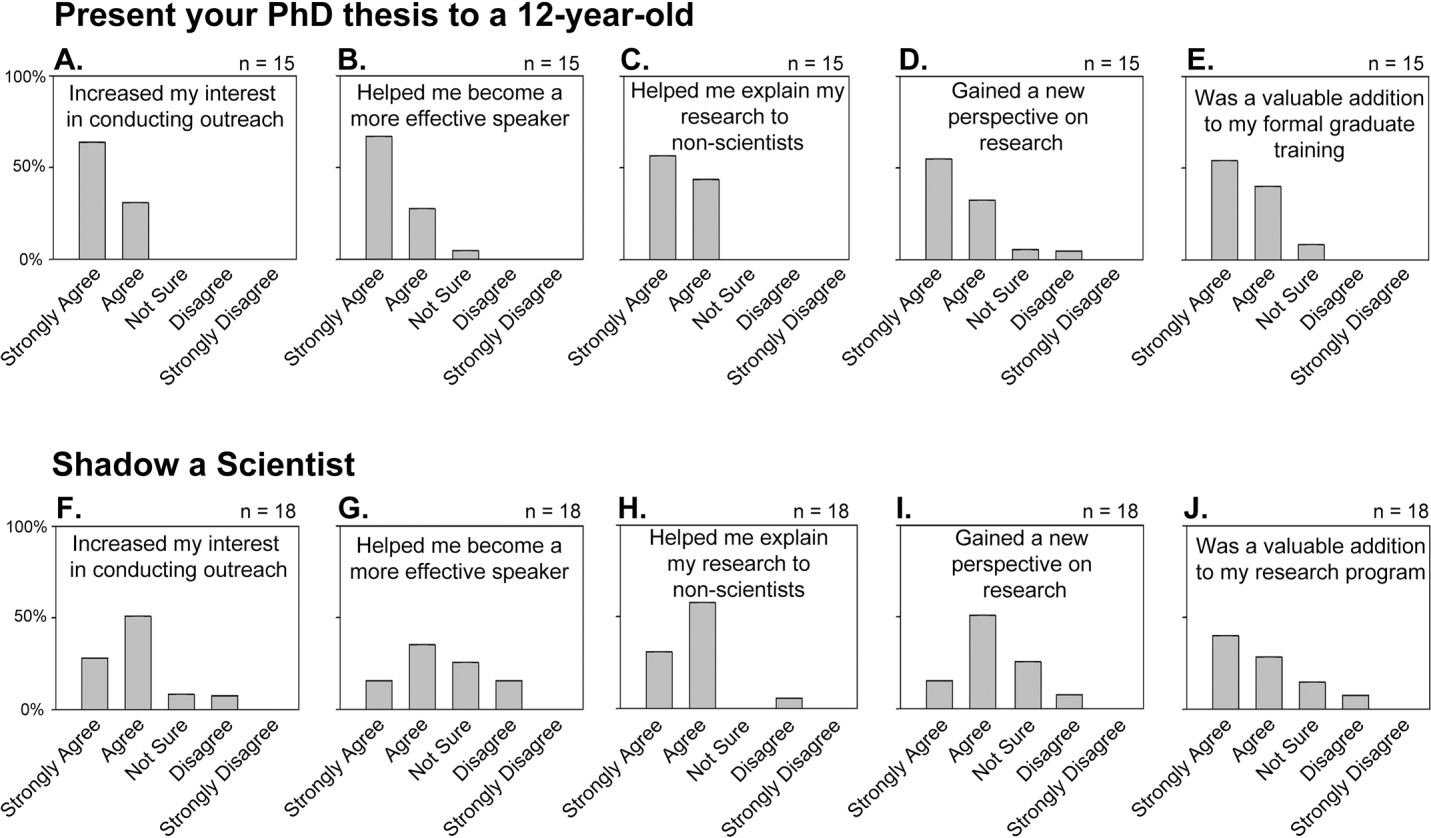
Survey responses of scientist participants. (A–E) The “Present Your PhD Thesis to a 12-Year-Old” program (*n* = 15) and (F–J) the “Shadow a Scientist” program (*n* = 18).

Almost all of the survey participants indicated that these programs improved their ability to explain their research (Fig 1C and 1H). However, the survey results indicate that the “Present Your PhD Thesis to a 12-Year-Old” program was more effective at improving participants’ scientific speaking skills (Fig 1B and 1G). This might be an expected outcome considering that the graduate students in this program spend time and effort preparing a presentation of a simplified version of their research. Additionally, scientists in this program may give many presentations, allowing them to modify their slides as well as their presentation delivery over time. Almost all of the participants in both programs indicated that the programs were valuable and provided them new perspectives on their research (Fig 1D and 1E and 1I and 1J).

**Fig 2:**
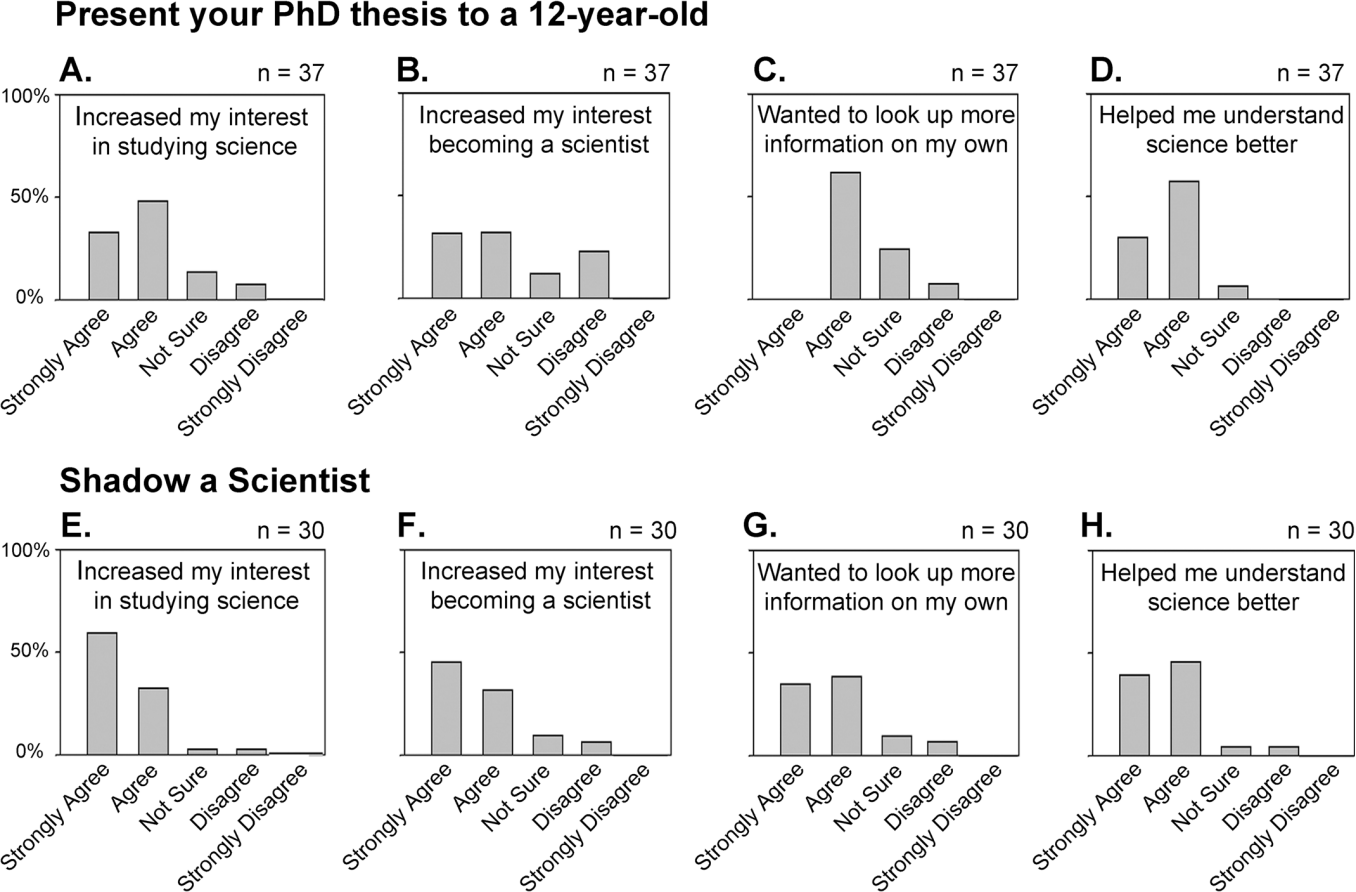
Survey responses of student participants. (A–D) The “Present Your PhD Thesis to a 12-Year-Old” program (*n* = 37) and (E–H) the “Shadow a Scientist” program (*n* = 31).

The surveys for the “Present Your PhD Thesis to a 12-Year-Old Program” were offered to students at an area magnet middle school with a science focus. Approximately 87% of the students responded that the presentations helped them to understand science better, while only 47% of the students indicated that the presentations made them want to look up more information on their own (Fig 2C and 2D). Perhaps more importantly, 70% of the students indicated that the program increased their interest in science, and 60% of the students responded that the program increased their interest in becoming a scientist (Fig 2A and 2B).

For the Shadow a Scientist program surveys, approximately 90% of the responding students indicated that they had a better understanding of science, and 75% had an increased interest in looking up more information on their own (Fig 2G and 2H). More than 80% of the students responded that participating in the program increased their interest in studying science and becoming scientists (Fig 2E and 2F). Based on the comments section of the surveys from Shadow a Scientist participants, some of the more memorable experiences that students reported while shadowing biologists include the following: learning about and even performing gel electrophoresis; examining cells using an electron microscope; and learning about telescopes and supercomputers from astronomers and computer scientists, respectively. The results from both programs show they seem to have a positive effect on students’ interest in science (Fig 2A and 2E). Overall, the student responses were slightly more positive for the Shadow a Scientist program, and this may be due to the longer, more personal interaction with a scientist that students receive in this program (Fig 2E–2H).

We performed statistical analyses comparing the results of the surveys between the two programs and generated *p*-values using the Wilcoxon rank-sum test as implemented in the exactRankTests library of R. Corrections for multiple testing were performed using the Dunn–Šidák method. The only two statistically significant differences (*p* < 0.05) in the responses were that scientists participating in the Present Your PhD to a 12-Year-Old program were much more likely to "strongly agree" that this program helped them to become more effective speakers and students participating in the Shadow a Scientist program were much more likely to “strongly agree” that this program made them want to look up more information on their own.

As with all program evaluations, our evaluation has limitations. For example, because our surveys were anonymous, we cannot determine whether students who participated in one program also participated in the other. We are also unable to track participants over time to see if they experience any longer-term outcomes. Another shortcoming is that our evaluation relied on self-reports of scientists’ gains in communication skills and student reports of their understanding of science. Meta-analytic work by Falchikov and Boud demonstrates that self-reports of knowledge or skill gains may or may not correlate with performance on more direct measures of knowledge or skills [18]. To address this shortcoming in the future, we will use more direct measures of scientists’ development of communication skills (e.g., Sevian and Gonsalves) [16]. We will also use more established, valid, and reliable measures of students’ interest in science and science careers (e.g., Gibson and Chase) [19] to see if we see similar effects as those we report here.

## Replication of Outreach Programs

One responsibility as scientists is to effectively communicate research and scientific concepts to the general public [2]. The need for more interaction between scientists and the public is widely recognized [11,20]. However, a study assessing scientists’ attitudes toward communication training found that scientists showed only moderate willingness to engage in science communication with the public in person [21]. Encouraging graduate students and research scientists to participate in K–12 STEM educational outreach programs is a direct way of addressing this issue. Here, we describe two relatively small, scientist-initiated and scientist-operated outreach programs that we believe are reproducible and scalable and can play valuable educational roles at any research institution and the local community they serve. Our two programs provide models for creating effective partnerships between scientists at universities and teachers and students at K–12 schools. They also help bridge the gap between graduate and postgraduate science communication training and K–12 educational experiences [10,22] by affording scientists the chance to educate young students about basic research while improving their science communication skills and sharing their enthusiasm for science. Our assessment indicates that, although not equal, both programs succeed to varying degrees in improving science communication and education.

In order to be successful in today’s extremely competitive field of science, scientists must dedicate a majority of their schedules to performing research. Most outreach programs described in the literature require a substantial time commitment from participating scientists and may even need a full-time person to organize and run. These programs are unique because they require a small input of time from the participating scientists and are inexpensive to implement yet yield documented positive benefits for the participants. These programs are an attractive option to address the acknowledged lack of science communication and outreach in universities. Thus, we call on other research scientists to replicate these programs at their universities, and we provide the information here as a guide to initiating these new outreach efforts.

## Supporting Information

S1 Fig Presentation rubric for middle school students’ evaluation of PhD thesis presentations. (PDF)

S2 Fig (A) List of venues in which PhD thesis presentation programs have been developed and (B) a sample list of presentation titles.
(TIF)

## Abbreviations

NSF: National Science Foundation
OMB: Office of Management and Budget
STEM: science, technology, engineering and math
