# Supplementary material for: Science Educational Outreach Programs That Benefit Students and Scientists
Source: PLoS Biol. 2016 Feb 4;14(2):e1002368. doi: 10.1371/journal.pbio.1002368 (PMC4742226; doi:10.1371/journal.pbio.1002368)
Supplement: S1 Fig — (PDF) [file pbio.1002368.s001.pdf]

Presenter Name: Research Topic:

SHARE YOUR Ph.D. with a 12 YEAR OLD<br>PRESENTATION RUBRIC	
**Criteria**	**Scale**
**Clarity:**<br>Is the presentation well thought out? Is it clear why the presenter chose to answer this particular question?	0 1 2 3 4 5
**Content:**<br>Does the presenter identify their research questions and experiments clearly? Does the presenter include other relevant research/experiments that led to their own?	0 1 2 3 4 5
**Style/Delivery:**<br>Is the presenter enthusiastic? Does the presenter use your names and make eye contact during the presentation? Does the presenter pace the presentation well?	0 1 2 3 4 5
**Use of visual aids:**<br>Does the presenter place images, charts, animations, figures, and images well in the PowerPoint? Does the presenter describe each well?	0 1 2 3 4 5
**Integration of Knowledge:**<br>Does the presenter describe why their research is important? Does the presenter identify future research related to their results?	0 1 2 3 4 5
**Ability to answer questions:**<br>Does the presenter understand questions that are being asked and answer them at a level that makes sense to you?	0 1 2 3 4 5

3 Things the presenter did well	3 Things the presenter could improve on	Questions I have…
+	Δ	?’s
