# Supplementary figures and images for: Science Educational Outreach Programs That Benefit Students and Scientists
Source: PLoS Biol. 2016 Feb 4;14(2):e1002368. doi: 10.1371/journal.pbio.1002368 (PMC4742226; doi:10.1371/journal.pbio.1002368)

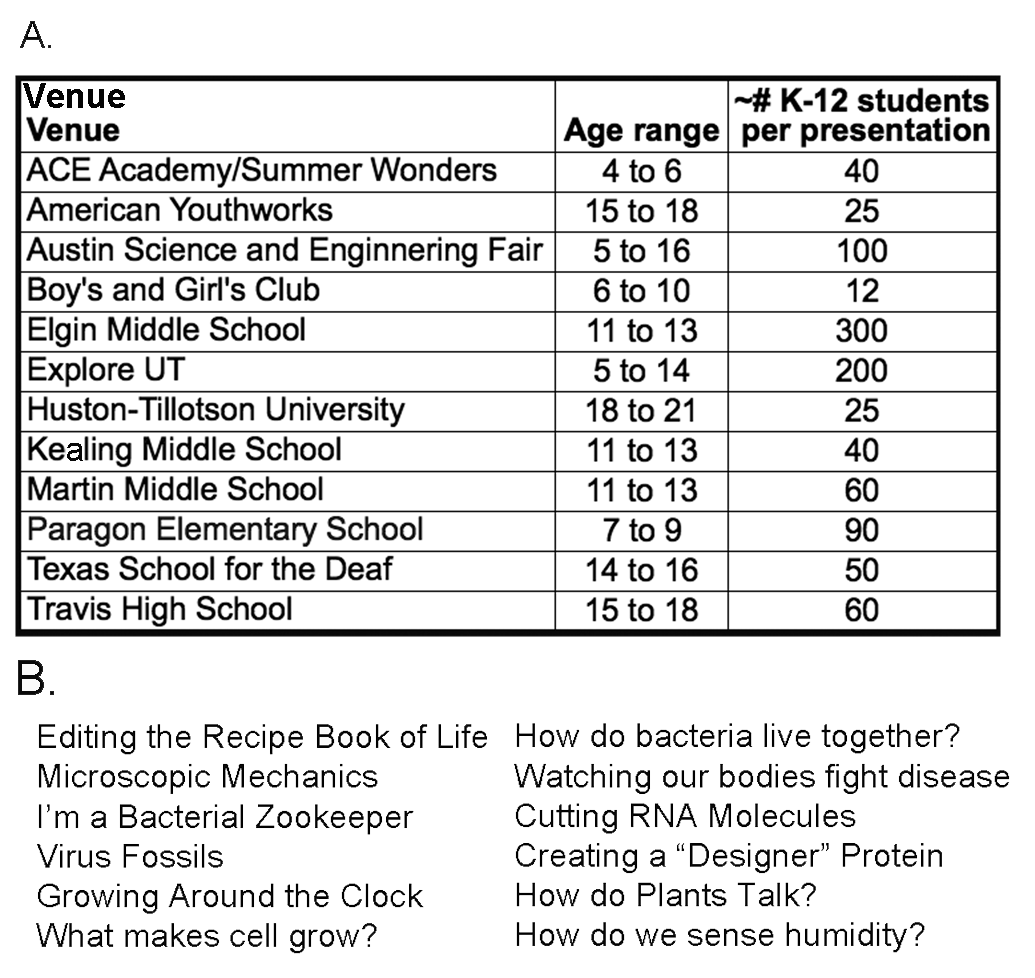

Supplement: S2 Fig — (A) List of venues in which PhD thesis presentation programs have been developed and (B) a sample list of presentation titles. (TIF) [file pbio.1002368.s002.tif]
